# Case Report: High-dose steroid and IVIG successful treatment in a case of COVID-19-associated autoimmune encephalitis: a literature review

**DOI:** 10.3389/fimmu.2023.1240089

**Published:** 2023-09-22

**Authors:** Chi-Hung Liu, Li-Chung Chiu, Chih-Chun Lee, Tien-Ming Chan

**Affiliations:** ^1^ Department of Medical Education, Chang Gung Memorial Hospital, Linkou Branch, Taoyuan, Taiwan; ^2^ Department of Thoracic Medicine, Chang Gung Memorial Hospital, Chang Gung University College of Medicine, Taoyuan, Taiwan; ^3^ Department of Medical Education, Chang Gung Memorial Hospital, Keelung Branch, Keelung, Taiwan; ^4^ Division of Rheumatology, Allergy, and Immunology, Department of Internal Medicine, Chang Gung Memorial Hospital, and Chang Gung University, Taoyuan, Taiwan

**Keywords:** autoimmune encephalitis, IVIG (intravenous immunoglobulin) administration, high-dose steroid therapy, COVID-19, diagnosis

## Abstract

Autoimmune encephalitis is a rare but critical complication of COVID-19. The management of COVID-19-associated autoimmune encephalitis includes the use of steroids, intravenous immunoglobulin (IVIG), plasmapheresis, and monoclonal antibody therapy. This study presented a patient with critical COVID-19 autoimmune encephalitis who rapidly recovered after the initiation of corticosteroids and IVIG therapy. This study reviewed the current literature on the pathophysiological mechanisms, diagnosis, and management of COVID-19-associated autoimmune encephalitis.

## 1 Introduction

As of March 2023, according to the World Health Organization, coronavirus disease 2019 (COVID-19), caused by severe acute respiratory syndrome coronavirus 2 (SARS-CoV-2), has afflicted more than 760 million people globally. Although most patients with COVID-19 had mild symptoms and recovered from the disease, COVID-19 was reported to induce autoimmune disorders in various organs (1), including autoimmune hepatitis, Guillain–Barre syndrome, and autoimmune encephalitis (2, 3). These conditions are associated with high mortality, and poor prognosis can be unrelated to the severity of infection (e.g., respiratory symptoms) (4). Autoimmune encephalitis is one of the immune-mediated disorders documented to occur with COVID-19. The most common symptoms include ataxia, hallucination, seizures, and memory deficit (5, 6).

## 2 Case report

A 22-year-old man who was diagnosed with COVID-19 on May 31, 2022, was admitted in July 2022 with complaints of fever for a week, headache for months, general weakness, neck pain, nausea and vomiting, and urinary retention. At the initial evaluation, he had a temperature of 38.8°C with clear consciousness (E4V5M6) and had no meningeal signs. He had no underlying systemic diseases and no history of alcohol and cigarette use. Laboratory data were normal, except for hyponatremia at 128 mEq/L (135–145 mEq/L) and hypokalemia at 3.0 mEq/L (3.5–4.5 mEq/L). The serological test showed equivocal MYCO-IgG (129.7 U/mL), and he was suspected of *Mycoplasma pneumoniae* infection. Under the impression of mycoplasma pneumonia, therapeutic management was initiated with broad-spectrum antibiotics, acyclovir, and dexamethasone.

**Figure 1:**
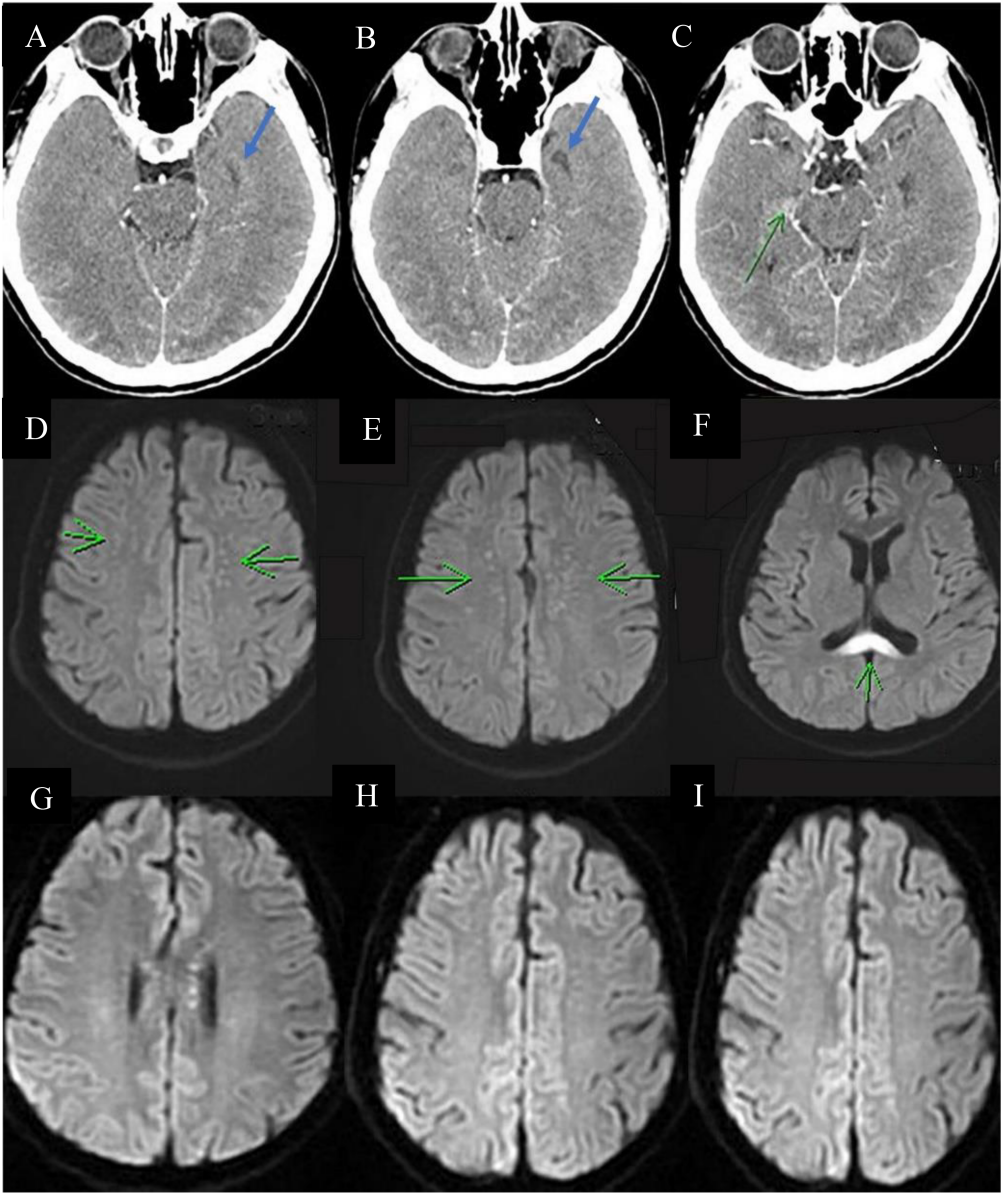
Serial images of the brain. Brain CT **(A–C)** showing a small hypodense lesion (blue arrow) and a small hyperdense lesion (green arrow) at the left superior frontal lobe and right temporal lobe, respectively. Brain MRI showing multiple tiny foci in bilateral centrum semiovale (green arrow in **(D, E)**) and diffuse restriction at the splenium of the corpus callosum [green arrow in **(F)**]. These findings are compatible with cytotoxic lesions of the corpus callosum, which indicate encephalitis. **(G–I)** Decreased diffusion restriction at the splenium of the corpus callosum and decreased abundance of multiple tiny foci in the bilateral centrum semiovale, suggesting regression of inflammation and CLOCCs.

**Figure 2:**
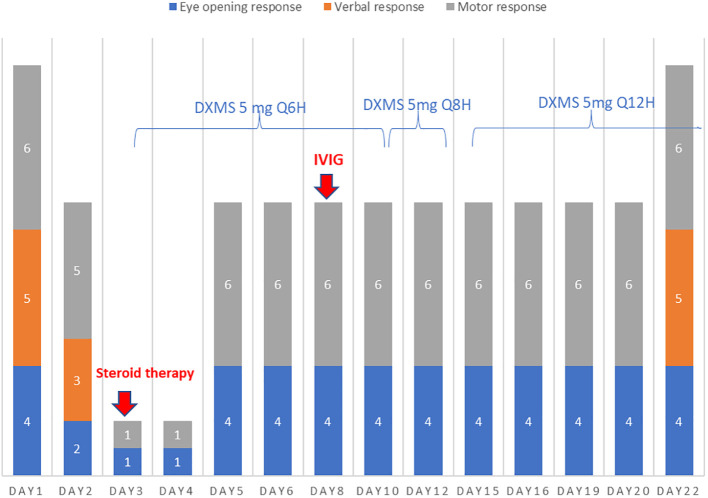
On days 3–22, the patient’s verbal response could not be evaluated because of intubation. On day 3, steroid therapy with dexamethasone (DXMS) at a dose of 5 mg every 6 h was initiated and gradually tapered to every 8 and 12 h on days 10 and 12, respectively. The steroid therapy was continued until discharge, totaling 41 days. In addition, intravenous immunoglobulin (IVIG) therapy was started at a dose of 0.5 g/kg/day on day 7 and continued for 5 days. The patient’s clinical response to the treatments is shown in the subsequent panels.

Over the next 2 days, the patient developed neurological symptoms, including disorientation and hallucination. Brain computed tomography (CT) showed a hypodense lesion at the left superior frontal lobe, and a small hyperdense lesion at the right temporal lobe, which indicated brain inflammation or acute infarction (**Figures 1A–C**). The patient’s condition further deteriorated, with a decreased level of consciousness (E1V1M1) and quadriplegia (upper limb muscle power graded as bilateral 0 and 1 at the proximal and distal sites, respectively, with the same degree of weakness observed in the lower limbs). Accordingly, the patient required intubation because of respiratory failure. Brain magnetic resonance imaging (MRI) revealed cytotoxic lesions of the corpus callosum (CLOCCs) (**Figures 1D–F**) and based on the Graus criteria (**Table 1**), the patient was diagnosed with encephalitis, and dexamethasone therapy was started (5 mg Q12H) on day 3. Within 3 days of steroid therapy, the patient’s level of consciousness improved to E4VeM6 (**Figure 2**).

**Table 1:** Graus diagnostic criteria for possible autoimmune encephalitis (7).

Criteria (7)	Our patient
1. Rapid progression (within 3 months) of CNS symptoms*	Consciousness changes within 3 days (Yes)
2. Exclusion of well-defined syndromes of autoimmune encephalitis^†^	(Yes)
3. Absence of well-characterized autoantibodies in the serum and CSF, and at least two of the following criteria: - MRI abnormalities suggestive of autoimmune encephalitis - CSF pleocytosis, CSF-specific oligoclonal bands or elevated CSF IgG index, or both - Brain biopsy showing inflammatory infiltration and excluding other disorders (e.g., tumor)	Neuroimmunology test showed negative finding (Yes) Cytotoxic lesion of the corpus callosum (Yes) CSF leukocyte of 195/mm^3^ (Yes) Brain biopsy was not performed. (No)
4. Reasonable exclusion of alternative causes	The differential diagnoses of autoimmune encephalitis were excluded (e.g., infections, tumors, Creutzfeldt–Jakob disease, post-epilepsy symptoms, and metabolic encephalopathy. (Yes)

* CNS symptoms include working memory deficits (short-term memory loss), altered mental status, or psychiatric symptoms.

† (e.g., typical limbic encephalitis, Bickerstaff’s brainstem encephalitis, and acute disseminated encephalomyelitis)

Graus criteria for evaluating probable autoimmune encephalitis with negative autoantibody results (adapted from *Lancet Neurology* 2016).

## 3 Glasgow coma scale and treatment

Cerebrospinal fluid (CSF) analysis (**Table 2**) showed a high leukocyte count at 195/µL (<5/μL) with 57% lymphocyte, elevated lactate at 56.8 (10–22) mg/dL, and total protein at 178.2 (15–45) mg/dL; however, CSF polymerase chain reaction and neuroimmunology tests including tests for common infectious pathogens (i.e., *E. coli* K1, *H. influenza*, cytomegalovirus, HSV-1, HSV-2, HHV-6, and VZV) and common autoantibodies for autoimmune encephalitis (i.e., anti-N-methyl-d-aspartate [NMDAR] antibodies, GABA B receptor antibodies, leucine-rich glioma-inactivated 1 antibodies, anti-contactin-associated protein-like-2 (CASPR2R) antibodies, alpha-amino-3-hydroxy-5-methyl-4-isoxazolepropionic acid antibodies, and dipeptidyl-peptidase-like protein 6 antibodies) showed negative findings. On day 8 after admission, electroencephalography showed intermittent diffuse theta and delta waves, which indicated diffuse cerebral dysfunction. On the same day, the patient started a 5-day course of IVIG therapy at a dose of 0.5 g/kg/day. The power of upper limb muscles improved from 1/5 to 2/5, and the fever subsided on day 11. However, the power of bilateral lower limb muscles remained deficient (0/5). A repeat lumbar puncture was performed on day 14, which showed a decrease in the leukocyte count (from 195/µL to 23/µL, with 100% lymphocytes), as well as in lactate and total protein levels (from 56.8 to 25.6 mg/dL and from 178.2 to 72.0 mg/dL, respectively). No autoimmune antibodies were detected. Brain MRI revealed mild regression of encephalitis and CLOCCs (**Figures 1G–I**). Nerve conduction velocity was performed on day 15 due to poor improvement in the power of lower limb muscles, which showed bilateral peroneal neuropathy with axon loss and mild denervation of the left first dorsal interosseous and biceps muscles. After two more days, the patient was successfully extubated and transferred to the general ward. On day 44, the patient was discharged with neurological sequelae, including decreased lower limb power (2/5) and urinary retention.

**Table 2:** Findings related to the cerebrospinal fluid throughout disease progression.

	Day 2 after admission	Day 14 after admission	Reference range
Gram staining	No bacteria seen	No bacteria seen	
Appearance	Clear	Clear	
Color	Colorless	Colorless	
Leukocyte count	195/µL	23/µL	<5/µL
RBC count	35/µL	9/µL	1/µL
Neutrophils	5%	0%	0–6%
Lymphocytes	57%	100%	40–80%
Monocytes	38%	0%	25–45%
Glucose	46 mg/dL	33 mg/dL	50–80mg/dL
Lactate	56.8 mg/dL	25.6 mg/dL	10–22mg/dL
Total protein	178.2 mg/dL	72.0 mg/dL	15–45mg/dL

The patient did not meet the diagnostic criteria for limbic encephalitis (8) or Bickerstaff’s brainstem encephalitis (9). No diagnostic criteria have been established for disseminated encephalomyelitis, and based on the brain MRI findings, the patient did not exhibit the characteristic features of disseminated encephalomyelitis (10, 11).

## 4 Discussion

This report presents a case of severe COVID-19-associated autoimmune encephalitis that improved following IVIG therapy. Encephalitis is a rare complication with a relatively high mortality rate in patients with COVID-19. Respiratory symptoms can be mild or even absent in affected individuals. Altered mental status, movement disorders, and seizures are the main domains of neurological symptoms in patients with COVID-19-associated autoimmune encephalitis. Similar to other types of autoimmune encephalitis, immunotherapy has shown promising clinical outcomes (12).

Three pathophysiological mechanisms of neurological complications are possible, namely, encephalitis, in COVID-19 (13, 14):

The first and most accepted mechanism is molecular mimicry triggered by COVID-19. In this theory, the activated host antibody identifies self-antigen and attacks various systems (15). Some common antineuronal autoantibodies are found in patients with COVID-19-associated autoimmune encephalitis (6, 16). include anti-NMDAR antibody, anti-glutamic acid decarboxylase antibody, anti-CASPR2 antibody, and anti-myelin oligodendrocyte glycoprotein. This pathophysiological mechanism can also explain other autoimmune diseases associated with COVID-19, including autoimmune hepatitis, Guillain–Barré syndrome, myelitis, acute disseminated encephalomyelitis, and rarely, autoimmune adrenalitis (17).

Second, cytokine storm due to the production of excessive amounts of pro-inflammatory cytokines, including tumor necrosis factor-α, interleukin (IL)-1, and IL-6, is another mechanism (18). According to a review article, one possible pathway is that cytokines in the peripheral circulation system impair the neurovascular endothelium and increase the permeability of the blood–brain barrier (BBB) (19, 20). If cytokine storm occurs, a hyperinflammatory state is formed, which leads to brain tissue damage and encephalitis. CSF analysis and serological tests such as D-dimer and C-reactive protein can provide evidence of this mechanism (21). The electroencephalogram pattern of autoimmune encephalitis sometimes shows a diffuse slowing wave indicative of systemic inflammation. It is worth mentioning that some biomarkers involved in inflammatory response may have an association with the severity of the disease and the overall prognosis. Rizzi et al. described IFN-γ-induced protein 10 (IP-10) and CRP could be prognostic markers in severe COVID-19 patients (22). Another research reported by Tonello et al. found that osteopontin (OPN) can be a promising biomarker to stratification COVID-19 severity (23).

Third, direct viral invasion of the central nervous system occurs (6, 18). The two main pathways of direct viral invasion include hematogenous spread and the trans-neural retrograde route (18, 19). In hematogenous spread, the virus may cross the BBB and invade the cerebral tissue by binding the angiotensin-converting enzyme II receptors of epithelial cells by the viral spike protein (24). SARS-CoV can also infect different myeloid cells such as leukocytes and can disseminate to different tissues, including the CNS (25). For the trans-neural retrograde pathway, the virus invades the olfactory neurons and utilizes the retrograde axon transportation to cross the cribriform plate and invade the CNS (26, 27). This route may play an important role in the occurrence of anosmia, one of the most common symptoms of COVID-19 (18, 26). According to the three mechanisms described above, autoimmune encephalitis may be present without CSF autoantibodies. Therefore, clinicians should not rule out autoimmune encephalitis even if autoantibodies are negative. In our patient with no clinical and laboratory evidence of active COVID-19 infection, autoantibodies for autoimmune encephalitis also had a negative result; thus, the cytokine storm may be a more appropriate explanation to this case. Still, no established guidelines have been widely utilized for the treatment of COVID-19-associated autoimmune encephalitis because of the limited number of cases. However, intravenous administrations of high doses of corticosteroids and immunoglobulins are the most widely used treatment. In 2020, Pilotto et al. reported a 60-year-old man with autoantibody-negative encephalitis treated with high-dose intravenous administration of corticosteroid (methylprednisolone 1 g per day) for 5 days (10). The patient’s consciousness improved (from akinetic mutism to alert) after the first cycle of steroid infusion and was discharged with a normal neurological examination. In the present report, we present a case of severe COVID-19-associated autoimmune encephalitis that improved following the administration of IVIG. Encephalitis is a rare complication with a relatively high mortality rate in patients with COVID-19. Respiratory symptoms can be mild or even absent. Altered mental status, movement disorders, and seizures are the main domains of neurological symptoms in patients with COVID-19-associated autoimmune encephalitis. Similar to other types of autoimmune encephalitis, immunotherapy has shown promising clinical outcomes (12).

Another case report with a review of the literature presented a case of COVID-19-associated anti-VGKC limbic encephalitis that recovered after treatment with corticosteroids. The case involves a 67-year-old woman with CNS symptoms including truncal ataxia, motor aphasia, and cognitive impairment. She had progressive improvement after starting on intravenous dexamethasone therapy and was finally discharged with nearly full recovery. The article also recommended corticosteroids as first-line therapy and be started immediately after diagnosis (16).

Plasmapheresis was also reported to be effective. In 2020, Dogan et al. conducted the first case series to describe the effect of plasmapheresis in COVID-19-associated autoimmune encephalitis (28). In the study, six cases of COVID-19-associated autoimmune encephalitis received plasmapheresis with albumin, and the clinical status, laboratory data (especially serum ferritin levels), and brain MRI during the disease course were recorded in the ICU setting. One of the patients had worsened clinical status, and others regained consciousness and could be extubated after 1–3 cycles of plasmapheresis. The laboratory data (especially serum ferritin levels) and brain MRI also suggested regression of encephalitis (28).

In the same year, Cao et al. performed a case series of five patients who received methylprednisolone intravenously 1 g/day for 5–10 days and plasma exchange (29). Three patients showed dramatic neurological improvements with improved consciousness within a week. Monoclonal antibodies such as rituximab can also be a treatment choice. Case reports have demonstrated the effectiveness of rituximab. One of the patients with severe neurological symptoms had generalized seizures and hallucinations that progressively improved after rituximab administration (30). A summary of the mentioned articles and the conclusion of the treatment choice are briefly described in **Table 3**.

**Table 3:** Characteristics of included cases and the summary of treatment to COVID-19-associated autoimmune encephalitis.

Authors	Study designs	Number of patients	General and respiratory symptoms	Neurological symptoms	Immunomodulatory treatments	Outcome
Pilloto et al. 2020 (11)	Case report	1	Cough, fever, normal-oxygen saturation	Confusion, aphasia, akinetic- mutism	Methylprednisolone	Discharge with no sequalae
Stoian et al. 2022 (31)	Case report systematic review of literature	1	Fever, cough, desaturation	Truncal ataxia, aphasia	Dexamethasone (2*8mg)/day Mycophenolate-mofetil	Fully recovered
Dogan et al. 2020 (16)	Case series	6	ARDS (intubated in ICU)	Agitated- delirium, loss of consciousness	Plasmapheresis with albumin	4 patients regained consciousness and discharged from ICU. The other 2 were still in ICU and one of them died.
Cao et al. 2020 (28)	Case series	5	ARDS	Impaired consciousness	Plasma exchange, corticosteroid	3 patients with significant improvement, the other 2 patients died.
Alvarez Bravo et al. 2021 (30)	Case report	1	Fever	Visual- hallucination, dysarthria, generalized- tonic-clonic seizure	Methylprednisolone (1 g/day), IVIG (1 mg/kg) and Rituximab	Discharged with cognitive sequalae such as hypoprosexia enrolled in rehabilitation

Common treatment of COVID-19-associated autoimmune encephalitis (15, 17–20)

First-line 1. Glucocorticoids (Usually methylprednisolone) 2. IVIG 3. Plasmapheresis Second line* 1. Rituximab 4.*The second line therapy should be considered if patients’ condition did not improve after the first-line therapy for 2–4 weeks (19).

This case report highlights the effectiveness of combined corticosteroid and IVIG therapy in managing COVID-19-associated autoantibody-negative autoimmune encephalitis. Previous reports have documented the success of IVIG or corticosteroid treatments in COVID-19-associated autoimmune encephalitis. However, clinical evidence on the efficacy and safety of combining IVIG and corticosteroids remains limited. In this instance, our patient experienced a favorable outcome following the administration of the combined therapy. In conclusion, despite limited data and lack of guidelines regarding the use of high-dose steroids and IVIG, this therapeutic approach may still prove beneficial for patients with COVID-19-associated autoimmune encephalitis. Consequently, we propose that the incorporation of a combined regimen of IVIG and high-dose corticosteroids could potentially prove advantageous for patients diagnosed with autoimmune encephalitis, particularly in instances where there is a concomitant COVID-19 association; however further studies are required to validate this in detail.

## Data availability statement

The original contributions presented in the study are included in the article/supplementary material. Further inquiries can be directed to the corresponding author.

## Ethics statement

Written informed consent was obtained from the participant/patient for the publication of this case report.

## Author contributions

Conceptualization, T-MC; methodology, T-MC; software, T-MC; validation, T-MC; formal analysis, T-MC; investigation, T-MC; resources, T-MC; data curation, C-HL and T-MC; writing—original draft preparation, C-HL and T-MC; writing—review and editing, C-HL, C-CL, and T-MC; visualization, T-MC; supervision, T-MC; project administration, T-MC; funding acquisition, T-MC. All authors contributed to the article and approved the submitted version.
